# A constitutive model for developing blood clots with various compositions and their nonlinear viscoelastic behavior

**DOI:** 10.1007/s10237-015-0686-9

**Published:** 2015-06-05

**Authors:** Thomas H. S. van Kempen, Wouter P. Donders, Frans N. van de Vosse, Gerrit W. M. Peters

**Affiliations:** Department of Biomedical Engineering, Eindhoven University of Technology, PO Box 513, 5600MB Eindhoven, The Netherlands; Department of Biomedical Engineering, School for Mental Health and Neuroscience, Maastricht University, Maastricht, The Netherlands; Department of Mechanical Engineering, Eindhoven University of Technology, Eindhoven, The Netherlands

**Keywords:** Blood clotting, Mechanical modeling, Large amplitude oscillatory shear (LAOS), Sensitivity analysis

## Abstract

The mechanical properties determine to a large extent the functioning of a blood clot. These properties depend on the composition of the clot and have been related to many diseases. However, the various involved components and their complex interactions make it difficult at this stage to fully understand and predict properties as a function of the components. Therefore, in this study, a constitutive model is developed that describes the viscoelastic behavior of blood clots with various compositions. Hereto, clots are formed from whole blood, platelet-rich plasma and platelet-poor plasma to study the influence of red blood cells, platelets and fibrin, respectively. Rheological experiments are performed to probe the mechanical behavior of the clots during their formation. The nonlinear viscoelastic behavior of the mature clots is characterized using a large amplitude oscillatory shear deformation. The model is based on a generalized Maxwell model that accurately describes the results for the different rheological experiments by making the moduli and viscosities a function of time and the past and current deformation. Using the same model with different parameter values enables a description of clots with different compositions. A sensitivity analysis is applied to study the influence of parameter variations on the model output. The relative simplicity and flexibility make the model suitable for numerical simulations of blood clots and other materials showing similar behavior.

**Electronic supplementary material:**

The online version of this article (doi:10.1007/s10237-015-0686-9) contains supplementary material, which is available to authorized users.

## Introduction

Blood clot formation is the main process that prevents blood loss after a vascular injury. However, a malfunction of this process can have dramatic consequences. A clot that is too strong can occlude a blood vessel leading e.g., to thrombosis or ischemia of peripheral tissue. On the other hand, a clot that closes the injury insufficiently leads to excessive bleeding. This shows that the mechanical properties of the blood clot are a major factor for its functioning (Jackson 2011; Tran et al. 2013).

The mechanical properties of the blood clot are determined by its structural composition (Jen and McIntire 1982; Shah and Janmey 1997; Gersh et al. 2009; Undas and Ariëns 2011). Clot formation starts when the injured vessel wall or another thrombogenic surface activates platelets present in the blood. The activated platelets form a plug that provisionally closes the injury (de Groot et al. 2012). Simultaneously, fibrinogen, usually an inactive protein in blood plasma, is converted into fibrin that subsequently forms a network of fibers that gives strength to the platelet plug (Weisel 2008). The fibrin network surrounds the platelets that contract and pull on the fibers (Jen and McIntire 1982; Lam et al. 2011). Furthermore, red blood cells that occupy about 45 % of the blood volume become entrapped in the clot (Gersh et al. 2009).

The many components that play a role, and their interactions, give the blood clot its complex mechanical properties that are important for a proper functioning (Jackson 2011; Tran et al. 2013). This behavior includes nonlinear viscoelasticity and depends on the deformation history that the clot has experienced (Shah and Janmey 1997; Münster et al. 2013; van Kempen et al. 2015).

The mechanical properties of blood clots in relation to their composition have been studied experimentally (Dintenfass 1971; Evans et al. 2006). Various rheological experiments have shown that the active contraction of platelets within the fibrin network gives clots a higher stiffness (Glover et al. 1975; Kirkpatrick et al. 1979; Jen and McIntire 1982; Shah and Janmey 1997). On the other hand, the presence of red blood cells leads to clots with a lower stiffness (Kaibara and Fukada 1970; Tynngård et al. 2006; Gersh et al. 2009). It has been shown that the viscoelastic properties of the blood clot can be used to distinguish healthy and diseased blood (Isogai et al. 1973; Fukada et al. 1984). The nonlinear viscoelastic properties of the clots are expected to be important for its functioning and have been studied experimentally by subjecting them to increasing deformations (Fukada et al. 1984; Burghardt et al. 1995; Shah and Janmey 1997; Riha et al. 1999). However, these experiments are usually analyzed in terms of the linear viscoelastic moduli, which is insufficient to fully understand this complex behavior (Hyun et al. 2011). Recently it has been shown that multiple deformation cycles lead to the loss of structural integrity of the clot (Münster et al. 2013; van Kempen et al. 2015).

To better understand the mechanical properties of the clot in relation to its structure, models should be developed that describe these properties as a function of the components (Xu et al. 2011). Furthermore, such constitutive models are necessary for numerical simulations of blood clot deformation due to blood flow (Storti et al. 2014). Such simulations have already shown to provide valuable insights (Anand et al. 2003; Bodnár and Sequeira 2008), but lack the correct constitutive law that governs to a large extent the outcome of such simulations. Therefore, in this study, a constitutive model for the blood clot is developed.

Several models exist that describe the mechanical properties of the components of the blood clot (Xu et al. 2011). Most of the models focus on the coagulation cascade (Bodnár and Sequeira 2008; Anand et al. 2003) or the fibrin network (Riha et al. 1997; Storm et al. 2005; van Kempen et al. 2014, 2015). The linear viscoelastic properties of the blood clot have been modeled using ultrasound techniques (Viola et al. 2004; Schmitt et al. 2011). Models for the intraluminal thrombus in abdominal aortic aneurysms have been developed (van Dam et al. 2008; Karšaj and Humphrey 2009) that describe the mechanical properties of the clot during the long time scale of years. The goal of this study is to develop a model that describes the mechanical properties of the developing clot, with a typical time scale of hours. Furthermore, the model should describe the nonlinear viscoelastic properties of the clots and the loss of structural integrity due to the deformation history. Such a model that describes both the developing blood clot and its nonlinear viscoelastic behavior has to the best of our knowledge not been developed before. The application of the constitutive model is in the numerical simulations of blood clot formation to study (patho)physiological conditions (Storti et al. 2014) which sets a limitation for the complexity of the model.

In this study, the nonlinear viscoelastic properties are studied using a dedicated analysis of large amplitude oscillatory shear (LAOS) deformations, as recently applied to fibrin networks (van Kempen et al. 2015). Besides this nonlinear behavior, the linear viscoelastic properties are measured during the formation of the clots. Thus, the rheological results are used to develop a set of constitutive models for blood clot formation and for the linear and nonlinear viscoelastic behavior of the mature clots.

The constitutive model is based on experiments in which the mechanical properties of the blood clot are measured under well-controlled conditions. Measurements are performed on whole blood (WB), platelet-rich plasma (PRP) and platelet-poor plasma (PPP), to separate the contributions from red blood cells, platelets and fibrin, respectively. This makes the model also applicable for simulations of platelet plug formation without taking into account red blood cells. The formation of the blood clots is tracked by imposing a small oscillatory strain and measuring the resulting stress. After maturation, a range of frequencies for the oscillatory deformation is used to determine the linear viscoelastic behavior and subsequently the strain amplitude is increased to probe the nonlinear viscoelastic properties. The model is based on previously developed constitutive models for the fibrin network (van Kempen et al. 2014, 2015).

In the next section, the experimental protocol is introduced first, followed by the procedure used for the development of the constitutive model, the results obtained and a discussion of these results.

## Materials and methods

### Experimental methods

#### Blood preparation

In this study, blood clots are formed from porcine blood, obtained from Dutch landrace hybrid pigs slaughtered for human consumption in a local slaughterhouse. Following regular slaughterhouse procedures, the pigs are stunned using carbon dioxide and subsequently exsanguinated. Blood is collected in a beaker and quickly transferred to 50 ml tubes containing 5 ml sodium citrate (10.9 mM final concentration) to prevent clotting. A control tube, without citrate, is used to observe the clotting abilities of the blood. The blood is kept at room temperature and used for experiments within 4 h of collection. Whole blood (WB) is centrifuged (150*g*, 15 min) to obtain platelet-rich plasma (PRP, top layer). Platelet-poor plasma (PPP) is obtained by centrifugation of PRP (1000*g*, 15 min). Clotting is initiated by adding 10 ${\upmu l}$ clotting buffer (20 mM $\mathrm {CaCl_2}$, 1.0 U/ml human thrombin (Kordia, Leiden, the Netherlands), 20 mM HEPES, pH 7.4, final concentrations) to 140 ${\upmu l}$ WB, PRP or PPP and mixing gently with a pipette. For the WB samples, the concentrations of $\mathrm {CaCl_2}$ and thrombin are doubled. Control experiments with higher $\mathrm {CaCl_2}$ and thrombin concentrations gave the same results within experimental error. The addition of clotting buffer is defined as the start of the experiment, and results are corrected for the time between this moment and the start of the measurement.

#### Rheometry

The clotting sample is transferred quickly to the titanium cone–plate geometry (25 mm diameter, 0.02 rad cone angle) of an ARES rheometer (Rheometric Scientific, USA). Measurements are performed at 39 $^{\circ }\mathrm {C}$, and a layer of mineral oil is applied at the sample edge to minimize evaporation and surface effects. The rheological measurement consists of three parts. First, the formation of the clot is followed by imposing a small oscillatory shear deformation (frequency 1 Hz, strain amplitude 0.01) for 30 min. After this period, the viscoelastic moduli are steady and the viscoelastic behavior of the clots is studied by performing a frequency sweep. The frequency of the oscillation is increased from 0.63 to 63 rad/s, while the strain amplitude is maintained at 0.01. In the final part of the measurement, the nonlinear viscoelastic properties of the clots are studied by imposing a large amplitude oscillatory shear (LAOS) deformation. The strain amplitude is increased from 0.01 to 1 in 11 logarithmically spaced steps while maintaining the frequency at 1 Hz, followed by a strain amplitude of 0.01. Each strain amplitude is held for 30 s. The strain during the LAOS experiment is shown in Fig. 4a. Control experiments showed that performing the frequency sweep before the strain sweep did not influence the outcome of the strain sweep.

During the entire measurement, the outputs of the rotation and torque signals from the rheometer are stored using an analog-to-digital converter (ADC) as described previously (Wilhelm 2002; van Kempen et al. 2015). These signals are converted to strain and stress and are used to study the mechanical behavior of the clots during the LAOS deformation. The nonlinear data are analyzed by plotting the strain versus the stress in so-called Lissajous–Bowditch plots as used before (Ewoldt et al. 2008; van Kempen et al. 2015).

The three parts of the rheological measurement are used to develop the constitutive model.

### Model development

In this section, the viscoelastic model that describes the nonlinear viscoelastic properties of the blood clots is introduced. First, the required kinematics are discussed.

#### Kinematics

The deformation of a material from an undeformed state $\varOmega _0$ to a deformed state $\varOmega$ is described by the deformation gradient tensor $\varvec{F}$ (Hunter 1983; Macosko 1994). For a viscoelastic material, the deformation can be split into an elastic part, $\varvec{F}_\mathrm{e}$ and an inelastic part, $\varvec{F}_\mathrm{p}$ (Lee 1969),

1 $$\begin{aligned} \varvec{F} = \varvec{F}_\mathrm{e} \cdot \varvec{F}_\mathrm{p}. \end{aligned}$$

**Fig. 1:**
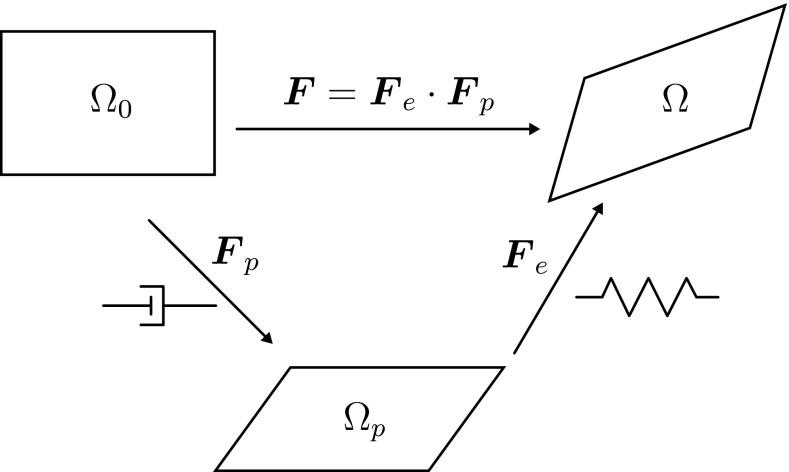
The deformation gradient tensor $\varvec{F}$ is split into an inelastic part $\varvec{F}_\mathrm{p}$ that brings the undeformed state $\varOmega _0$ to the stress-free configuration $\varOmega _\mathrm{p}$. The elastic part $\varvec{F}_\mathrm{e}$ transforms this state into the deformed state $\varOmega$

The inelastic part, $\varvec{F}_\mathrm{p}$, transforms the undeformed state to a relaxed, stress-free configuration $\varOmega _\mathrm{p}$. For a single mode, this fictitious configuration would be recovered instantaneously if all loads are removed from the material. The elastic part, $\varvec{F}_\mathrm{e}$, transforms this state $\varOmega _\mathrm{p}$ elastically into the deformed state $\varOmega$ (Fig. 1). Using the deformation gradient tensor, the Finger tensor, $\varvec{B}$, and its elastic equivalent, $\varvec{B}_\mathrm{e}$, are defined as,

2 $$\begin{aligned} \varvec{B} = \varvec{F} \cdot \varvec{F}^\mathrm{T} \quad \mathrm {and} \quad \varvec{B}_\mathrm{e} = \varvec{F}_\mathrm{e} \cdot \varvec{F}_\mathrm{e}^\mathrm{T}. \end{aligned}$$

The velocity gradient tensor is defined as,

3 $$\begin{aligned} \varvec{L} = \dot{\varvec{F}}\cdot \varvec{F}^{-1}, \end{aligned}$$

and can be split into an elastic and inelastic part,

4 $$\begin{aligned} \varvec{L} = \varvec{L}_\mathrm{e} + \varvec{L}_\mathrm{p}, \end{aligned}$$

with

5 $$\begin{aligned} \varvec{L}_\mathrm{e} = \dot{\varvec{F}_\mathrm{e}} \cdot \varvec{F}_\mathrm{e}^{-1} \quad \mathrm {and} \quad \varvec{L}_\mathrm{p} = \varvec{F}_\mathrm{e} \cdot \dot{\varvec{F}_\mathrm{p}} \cdot \varvec{F}_\mathrm{p}^{-1} \cdot \varvec{F}_\mathrm{e}^{-1}. \end{aligned}$$

Using the velocity gradient tensor $\varvec{L}$, the rate of deformation tensor $\varvec{D}$ is defined as,

6 $$\begin{aligned} \varvec{D} = \frac{1}{2}\,\left( \varvec{L} + \varvec{L}^\mathrm{T}\right) , \end{aligned}$$

and equivalently for the inelastic part,

7 $$\begin{aligned} \varvec{D}_\mathrm{p} = \frac{1}{2}\,\left( \varvec{L}_\mathrm{p} + \varvec{L}_\mathrm{p}^\mathrm{T}\right) . \end{aligned}$$

For numerical implementation, the inelastic right Cauchy-Green tensor is used,

8 $$\begin{aligned} \varvec{C}_\mathrm{p} = \varvec{F}_\mathrm{p}^\mathrm{T} \cdot \varvec{F}_\mathrm{p} = \varvec{F}^\mathrm{T} \cdot \varvec{B}_\mathrm{e}^{-1}\cdot \varvec{F}. \end{aligned}$$

Assuming spin-free inelastic deformation, the time derivative of $\varvec{C}_\mathrm{p}$ can be written as,

9 $$\begin{aligned} \dot{\varvec{C}_\mathrm{p}} = \varvec{C}_\mathrm{p} \cdot \varvec{F}^{-1} \cdot \varvec{D}_\mathrm{p} \cdot \varvec{F}, \end{aligned}$$

which will be used to update $\varvec{C}_\mathrm{p}$ during the time integration procedure.

More details on the kinematics of viscoelastic materials can be found elsewhere (Hunter 1983; Macosko 1994).

### Constitutive model

**Fig. 2:**
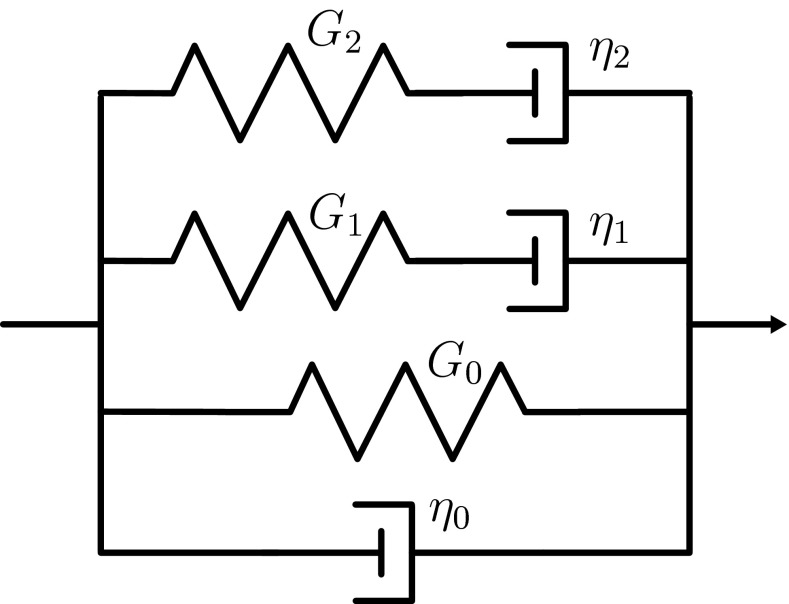
The constitutive model is based on a generalized Maxwell model that contains an equilibrium elastic $(G_0)$ and viscous $(\eta _0)$ mode parallel to two viscoelastic Maxwell modes with moduli $G_i$ and $\eta _i$

The constitutive model for the blood clot is inspired by previously developed models for the fibrin network (van Kempen et al. 2014, 2015) and abdominal aortic aneurysm thrombus (van Dam et al. 2008) and is based on a generalized multi-mode Maxwell model. The model can be represented by an elastic spring, a viscous dashpot and a number of Maxwell modes assembled in parallel (see Fig. 2). Two Maxwell modes are used in this study since this is sufficient to capture the frequency range explored. First, a simplified version of the model is introduced which is then extended to describe the viscoelastic, time-dependent and nonlinear properties of the clot. The same viscoelastic model is used for the clots formed from WB, PRP and PPP, but with different parameter values.

The model describes the stress $\varvec{\tau }$ as,

10 $$\begin{aligned} \varvec{\tau } = \varvec{\tau }_\mathrm{v} + \varvec{\tau }_\mathrm{e} + \sum _{i=1}^2 \varvec{\tau }_{\mathrm{ve},i}, \end{aligned}$$

with $\varvec{\tau }_\mathrm{v}, \varvec{\tau }_\mathrm{e}$ and $\varvec{\tau }_{\mathrm{ve},i}$ being the contribution to the stress from the viscous dashpot, elastic spring and Maxwell modes, respectively. Note that the model is defined in terms of the extra stress $\varvec{\tau }$ and that no hydrostatic pressure contribution is considered. This is a convenient description for the current incompressible, homogeneous situation. The elastic part of the viscoelastic modes is modeled as Neo–Hookean, and the stress is therefore given by,

11 $$\begin{aligned} \varvec{\tau }_{\mathrm{ve},i} = G_{i}\left( \varvec{B}_{\mathrm{e},i} - \varvec{I}\right) , \end{aligned}$$

with $G_i$ being the modulus of mode *i* and $B_{\mathrm{e},i}$ its elastic Finger tensor. The stress $\varvec{\tau }_{\mathrm{ve},i}$ is used to determine the inelastic rate of deformation tensor, $\varvec{D}_{\mathrm{p},i}$,

12 $$\begin{aligned} \varvec{D}_{\mathrm{p},i} = \frac{\varvec{\tau }_{\mathrm{ve},i}}{2\,\eta _i}, \end{aligned}$$

with $\eta _i$ being the viscosity of mode *i*.

The contribution to the stress from the elastic mode is modeled as Neo–Hookean and is given by,

13 $$\begin{aligned} \varvec{\tau }_{\mathrm{e}} = G_{0}\left( \varvec{B} - \varvec{I}\right) . \end{aligned}$$

The contribution of the viscous mode is separated into a contribution from the plasma with constant viscosity $\eta _\mathrm{p} = 4$ mPa s (Robertson et al. 2008), and a contribution from the clot $\eta _0$,

14 $$\begin{aligned} \varvec{\tau }_\mathrm{v} = 2\left( \eta _\mathrm{p} + \eta _0\right) \varvec{D}. \end{aligned}$$

The moduli $G_i$ and viscosities $\eta _i$, with $i=0,1,2$, describe the linear viscoelastic behavior of the clot. The properties of the developing clot are captured by making them a function of time. Subsequently, the nonlinear viscoelastic properties are included in the model by relating the moduli and viscosities to the current and past deformation.

### Frequency response

**Fig. 3:**
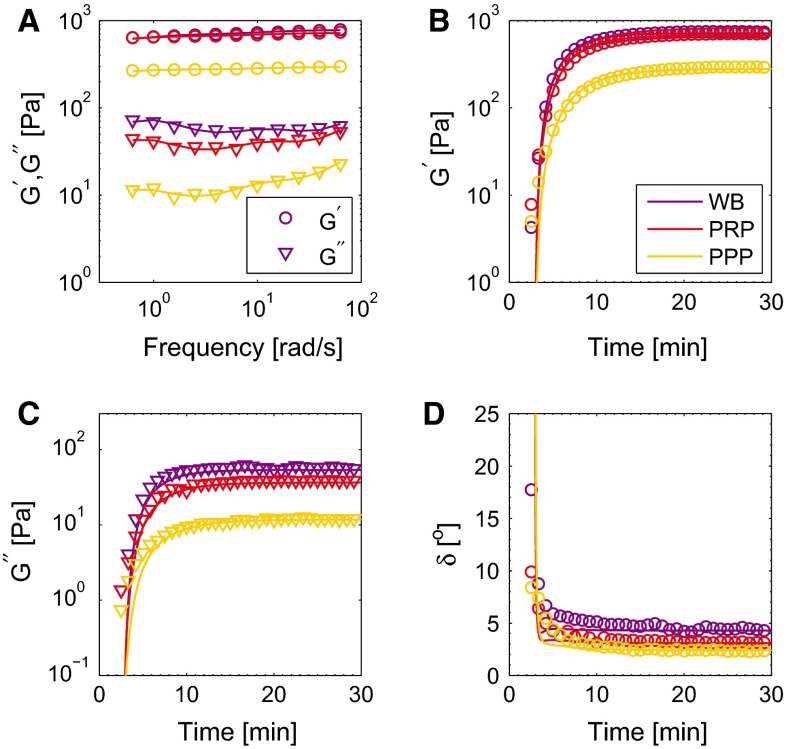
The viscoelastic moduli as measured in experiments (*symbols*) and described by the model (*lines*) for clots formed from WB, PRP and PPP. The elastic ($G^{\prime }$, *circle*) and viscous modulus ($G^{\prime \prime }$, *downward triangle*) show weak frequency dependencies (**a**). The elastic (**b**) and viscous moduli (**c**) increase during clot formation, but not at the same rate as shown by a decreasing phase angle ($\delta$, **d**)

The frequency response of the clots is used to determine values for the linear parameters $G_i$ and $\eta _i$ by comparing the elastic, $G^{\prime }$, and viscous, $G^{\prime \prime }$, moduli as measured in the experiment and described by the model (Macosko 1994),

15 $$\begin{aligned} G^{\prime }= & {} G_{0} + \displaystyle \sum _{i=1}^{2} G_i \frac{\left( \lambda _i\,\omega \right) ^2}{1 + \left( \lambda _i\,\omega \right) ^2}, \end{aligned}$$

16 $$\begin{aligned} G^{\prime \prime }= & {} \left( \eta _\mathrm{p} + \eta _0\right) \omega + \displaystyle \sum _{i=1}^{2} G_i \frac{\lambda _i\,\omega }{1 + \left( \lambda _i\,\omega \right) ^2}, \end{aligned}$$

with $\lambda _i = \eta _i/G_i$ being the relaxation time of mode *i* and $\omega$ the imposed frequency of the oscillatory deformation. Using the viscoelastic moduli, the frequency-dependent phase angle $\delta = \arctan {\left( G^{\prime \prime }/G^{\prime }\right) }$ can be defined. The viscoelastic model is able to capture the frequency response of the clots, as shown in Fig. 3a. This also shows that two viscoelastic modes give satisfying results.

### Blood clot formation

The mechanical properties during the formation of the clots are modeled by making the linear viscoelastic parameters a function of time,

17 $$\begin{aligned} G_i\left( t\right)= & {} f_\mathrm{e}\left( t\right) \, G_{i0}, \end{aligned}$$

18 $$\begin{aligned} \eta _i\left( t\right)= & {} f_\mathrm{v}\left( t\right) \, \eta _{i0}, \end{aligned}$$

where $G_{i0}$ and $\eta _{i0}$ are the values for the mature clots, obtained from the frequency response. The functions $f_\mathrm{e}\left( t\right)$ and $f_\mathrm{v}\left( t\right)$ describe the time dependency of the viscoelastic parameters. Inspired by a model previously developed to describe the mechanical properties of the developing fibrin network (van Kempen et al. 2014), an exponential increase in the moduli is chosen with a time constant $t_\mathrm{c}$. From the time-dependent viscoelastic moduli (Fig. 3 b, c), it is observed that they do not start to increase immediately but only after a delay. This delay period is attributed to the time needed for platelet activation and formation of protofibrils during the fibrin network formation (Glover et al. 1975; Jen and McIntire 1982) and is taken into account by shifting the exponential function in time with a delay time $t_0$. The mechanical properties of the blood clot change from fluid-like to solid-like behavior during the clot formation. Therefore, the ratio between the elastic and viscous moduli changes throughout the clot formation, as visible by a decreasing phase angle $\delta$ in Fig. 3d. This indicates that the elastic contribution to the stress increases more than to the viscous part and thus that the functions $f_\mathrm{e}\left( t\right)$ and $f_\mathrm{v}\left( t\right)$ cannot be the same. Based on a model for the maturing fibrin network (van Kempen et al. 2014), in which the elastic contribution increases quadratically with respect to the increase in the viscous modulus, the following equations are proposed,

19 $$\begin{aligned} f_\mathrm{e}\left( t\right)&= f_\mathrm{v}\left( t\right) ^{2}, \end{aligned}$$

20 $$\begin{aligned} f_\mathrm{v}\left( t\right)&= {\left\{ \begin{array}{ll} 0, &{} \text {if} \quad t \le t_0,\\ \left( 1 - e^{-\frac{\left( t-t_0\right) }{t_\mathrm{c}}}\right) , &{} \text {if} \quad t > t_0 \end{array}\right. } \end{aligned}$$

with $t_0$ being the delay time and $t_\mathrm{c}$ a time constant. As shown in Fig. 3, these functions enable a rather good description to the viscoelastic moduli in time for clots formed from WB, PRP and PPP with each of their own parameter values.

**Fig. 4:**
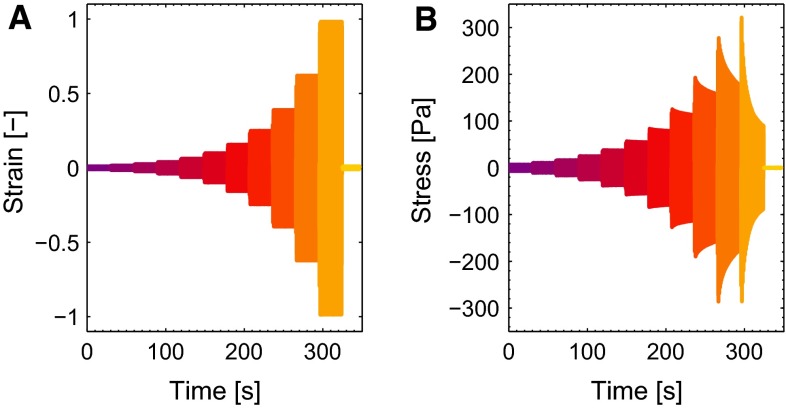
The imposed strain (**a**) and measured stress (**b**) during the LAOS experiments. The *different colors* indicate the interval with strain amplitudes increasing from 0.01 to 1

**Fig. 5:**
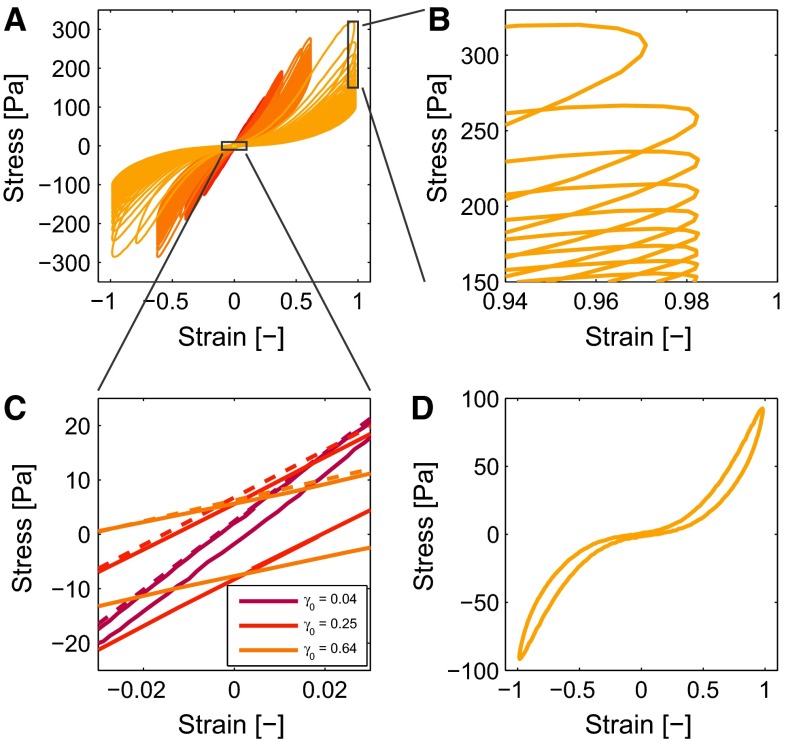
The nonlinear features are visible in the Lissajous–Bowditch plots (**a**). The softening effect can be observed by the decreasing maximal stresses (**b**) and the change of the slope around the low strain (**c**). This minimal strain modulus $G_m$ is estimated numerically and visualized with the *dashed lines*. Observing a singe loop shows the strain stiffening and the nonlinear viscous dissipation (**d**)

### Nonlinear viscoelastic behavior

The generalized Maxwell model is extended to describe the nonlinear viscoelastic behavior of the blood clots. The extension is a variation on a model previously used to describe the nonlinear properties of the fibrin network (van Kempen et al. 2015). The model development is explained using an illustrative example of a clot formed from WB. The same model, with different parameter values, is used to describe the behavior of clots formed from PRP and PPP, as will be shown in Sect. 3.

The results of a typical LAOS experiment, in which the strain amplitude increases in 11 steps from 0.01 to 1, are shown in Fig. 4.

Like previously described for a fibrin network (van Kempen et al. 2015), three nonlinear features are distinguished in the Lissajous–Bowditch plots (Fig. 5a): softening, strain stiffening and nonlinear viscous dissipation. The shear moduli and viscosities of the generalized Maxwell model are extended to incorporate these three features in the model, as discussed in detail next.

#### Softening

The stiffness of the clot decreases during multiple deformation cycles, as shown by the decreasing stress $\tau _0$ at maximal strain $\gamma _0$ (Fig. 5b). This is also visible by observing the slope of the Lissajous–Bowditch plots around zero strain $G_m$ (Fig. 5c), represented by a minimal strain modulus $G_m$,

21 $$\begin{aligned} G_m = \left. \frac{\partial \tau }{\partial \gamma }\right| _{\gamma = 0}. \end{aligned}$$

**Fig. 6:**
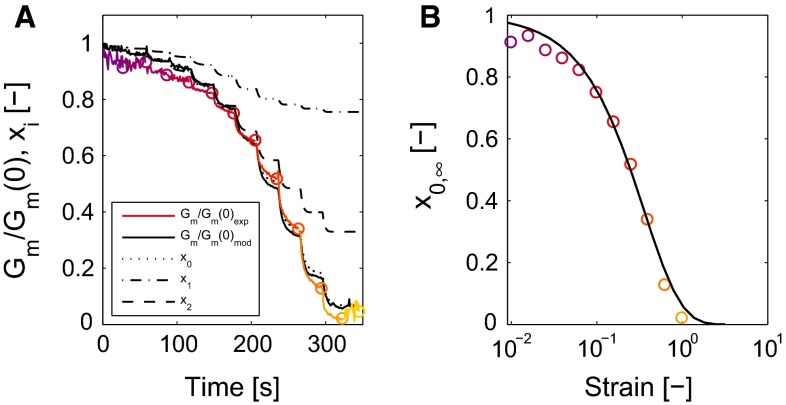
The normalized minimal strain modulus $G_m$ (**a**, *colored lines*, *colors* as in Fig. 4) decreases with increasing strain and is well described by the model (*solid black line*). The softening parameters, $x_i$, for the different modes *i* describe this behavior. Their behavior is described with the same kinetics, but is different for every mode because they experience a different elastic deformation (**a**, *dashed black lines*). The equilibrium values of $x_0$ are plotted versus the strain as found from $G_m$ (**b**, *circles*) and the model (*lines*)

This modulus, normalized by its initial value $G_m\left( 0\right)$, quantifies the softening behavior and is shown in Fig. 6a as a colored line. The value of $G_m$ decreases quickly when the strain amplitude increases, but subsequently levels off to a steady value. This softening behavior is incorporated in the model by decreasing the moduli using a state parameter $x_i$ for every mode that is a function of the deformation history. It is assumed that all moduli of the viscoelastic model are involved in this decrease. The modulus of every mode $G_i$ is related to a corresponding state parameter $x_i$,

22 $$\begin{aligned} G_i = x_i \, G_{i0}. \end{aligned}$$

An evolution equation for $x_i$ is used that describes the decrease in the modulus due to the deformation history. For reasons of simplicity the same evolution equation is used for all modes. Following the behavior of $G_m$, the equation should describe a decrease in $x_i$ with an increasing strain toward a value depending on this strain. The value of $x_i$ does not increase when the strain decreases, as shown by the value of $G_m$ during the last strain interval with amplitude of $\gamma _0 = 0.01$ (Fig. 6a). The following evolution equation is proposed,

23 $$\begin{aligned} \dot{x_i} = {\left\{ \begin{array}{ll} -c_x\,\left( x_i - x_{i,\infty }\right) &{} \text {if} \quad x_i>x_{i,\infty },\\ 0 &{} \text {if} \quad x_i \le x_{i,\infty }, \end{array}\right. } \end{aligned}$$

with $c_x$ being a fit parameter that describes the time scale of the decrease in $x_i$ and $x_{i,\infty }$ the level of $x_i$ corresponding to a certain strain,

24 $$\begin{aligned} x_{i,\infty } = e^{-a\,\sqrt{I_{B,i} - 3}}, \end{aligned}$$

with *a* being a fit parameter and $I_{B,i}$ the first invariant of the elastic Finger tensor of mode *i*. Note that for the equilibrium mode with modulus $G_0$, the elastic Finger tensor equals the total Finger tensor, $\varvec{B}_{\mathrm{e},0} = \varvec{B}$. For a simple shear deformation as used in the experiments, the function $\sqrt{I_{B,i} - 3}$ equals the shear strain $\gamma$ (Macosko 1994). To reduce the number of parameters in the model, the same parameter values are used for all state parameters $x_i$. Since every mode has its own elastic Finger tensor, $\varvec{B}_{\mathrm{e},i}$, the state parameter $x_i$ is different for every mode. Therefore, the contribution of every mode to the total stress will decrease at a different rate for every mode, as shown in Fig. 6a.

#### Strain stiffening

The stiffness of the clots increases with the strain, as shown by the increasing slope of the cycle in Fig. 5d. This strain-stiffening behavior is important for the functioning of the clot and should therefore be taken into account by the constitutive model (Shah and Janmey 1997; Riha et al. 1999). It is incorporated in the model by making the modulus of the equilibrium mode $G_0$ a function of the deformation,

25 $$\begin{aligned} G_0 = x_0 \, f_\mathrm{ss}\left( \varvec{B}\right) \, G_{00}. \end{aligned}$$

**Fig. 7:**
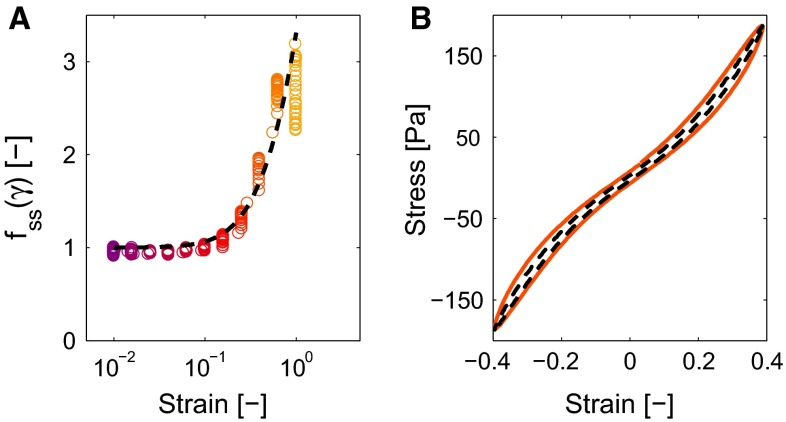
The function that describes the strain stiffening $f_\mathrm{ss}$ is a function of the strain. The model (*dashed line*) estimates the values obtained from the experimental data (*circles*). The *colors* correspond to the strain amplitudes in Fig. 4a. Visualizing a single loop **b** shows the nonlinear viscous dissipation as measured (*colored line*) and described by the model (*dashed line*)

To be able to extract this behavior from the Lissajous–Bowditch plots, the stress at maximal strain $\gamma _0$ during a cycle is used, because at this moment the contribution of the viscous mode vanishes (van Kempen et al. 2015). Therefore, the stress corresponding to this strain, $\tau _0$, is given by,

26 $$\begin{aligned} \tau _0 = x_0 \, f_\mathrm{ss}\left( \gamma _0\right) \, G_{00} \, \gamma _0 + \displaystyle \sum _{i=1}^2 \tau _{\mathrm{ve},i}\left( x_i, \gamma _0, \omega \right) , \end{aligned}$$

with $f_\mathrm{ss}\left( \gamma _0\right)$ being the function that describes the increasing stiffness that can be rewritten to,

27 $$\begin{aligned} f_\mathrm{ss}\left( \gamma _0\right) = \frac{\tau _0 - \displaystyle \sum _{i=1}^2 \tau _{\mathrm{ve},i}\left( x_i, \gamma _0\right) }{ x_0 \, G_{00} \,\gamma _0}, \end{aligned}$$

and can be obtained from the data as shown in Fig. 7a. Requirements for the function $f_\mathrm{ss}$ are that it equals one at low strain and is symmetric with respect to the strain. The function

28 $$\begin{aligned} f_\mathrm{ss}\left( I_{B}\right) = \left( 1 + k_1 \left( I_{B} - 3\right) \right) ^{n_1}, \end{aligned}$$

with $k_1$ and $n_1$ being fit parameters and $I_{B} - 3$ a symmetric measure for the strain, satisfies these criteria and gives satisfying results as shown in Fig. 7a.

#### Nonlinear viscous dissipation

The width of the Lissajous–Bowditch curves changes throughout a cycle, as shown in Fig. 7b. This indicates that the viscous dissipation increases with increasing strain. This behavior is incorporated in the model by making the viscosity of the equilibrium mode an increasing function of the deformation,

29 $$\begin{aligned} \eta _0 = f_{\mathrm{v}i}\left( \varvec{B}\right) \, \eta _{00}, \end{aligned}$$

with

30 $$\begin{aligned} f_{\mathrm{v}i}\left( I_{B}\right) = 1 + k_2\left( I_B - 3\right) , \end{aligned}$$

the function that describes the increasing viscosity as function of the deformation and $k_2$ being a fit parameter.

#### Model overview

An overview of the constitutive model is given by combining the equations from Sects. 2.3, 2.5 and 2.6.

31 $$\begin{aligned} \varvec{\tau }&= \varvec{\tau }_\mathrm{v} + \varvec{\tau }_\mathrm{e} + \sum _{i=1}^2 \varvec{\tau }_{\mathrm{ve},i}, \end{aligned}$$

32 $$\begin{aligned} \varvec{\tau }_\mathrm{v}&= 2\left( \eta _\mathrm{p} + f_\mathrm{v}\left( t\right) \,f_{\mathrm{v}i}\left( I_B\right) \, \eta _{00}\right) \varvec{D}, \end{aligned}$$

33 $$\begin{aligned} \varvec{\tau }_{\mathrm{e}}&= f_\mathrm{e}\left( t\right) \,x_0\, f_\mathrm{ss}\left( I_B\right) \, G_{00}\left( \varvec{B} - \varvec{I}\right) , \end{aligned}$$

34 $$\begin{aligned} \varvec{\tau }_{\mathrm{ve},i}&= x_i \, G_{i}\left( \varvec{B}_{\mathrm{e},i} - \varvec{I}\right) , \end{aligned}$$

35 $$\begin{aligned} \varvec{D}_{\mathrm{p},i}&= \varvec{\tau }_{\mathrm{ve},i}/2\,\eta _i, \end{aligned}$$

36 $$\begin{aligned} f_\mathrm{v}\left( t\right)&= {\left\{ \begin{array}{ll} 0, &{} \text {if} \quad t \le t_0,\\ \left( 1 - e^{-\frac{\left( t-t_0\right) }{t_\mathrm{c}}}\right) , &{} \text {if} \quad t > t_0, \end{array}\right. } \end{aligned}$$

37 $$\begin{aligned} f_\mathrm{e}\left( t\right)&= f_\mathrm{v}\left( t\right) ^{2}, \end{aligned}$$

38 $$\begin{aligned} f_{\mathrm{v}i}\left( I_{B}\right)&= 1 + k_2\left( I_B - 3\right) , \end{aligned}$$

39 $$\begin{aligned} f_\mathrm{ss}\left( I_{B}\right)&= \left( 1 + k_1 \left( I_{B} - 3\right) \right) ^{n_1}, \end{aligned}$$

40 $$\begin{aligned} \dot{x_i}&= {\left\{ \begin{array}{ll} -c_x\,\left( x_i - x_{i,\infty }\right) &{} \text {if} \quad x_i>x_{i,\infty },\\ 0 &{} \text {if} \quad x_i \le x_{i,\infty }, \end{array}\right. } \end{aligned}$$

41 $$\begin{aligned} x_{i,\infty }&= e^{-a\,\sqrt{I_{B,i} - 3}}. \end{aligned}$$

The model contains three moduli, $G_{i0}$, three viscosities $\eta _{i,0}$ and seven fit parameters, $t_0, t_\mathrm{c}, a, c_x, k_1, n_1$ and $k_2$, that are determined using a stepwise fitting procedure as described in the next section.

### Numerical procedures

The parameter values are found using the data from the rheometer experiments, in which a sinusoidal shear strain, $\gamma$, is imposed and the shear stress $\tau$ is measured. For this deformation, the deformation gradient tensor in time is given by (Macosko 1994),

42 $$\begin{aligned} \varvec{F}\left( t\right) = \begin{pmatrix} 1 &{} \gamma \left( t\right) &{} 0 \\ 0 &{} 1 &{} 0\\ 0 &{} 0 &{} 1 &{} \end{pmatrix}, \end{aligned}$$

which is used as an input for the constitutive model to determine the resulting stress tensor $\varvec{\tau }$ and its shear component $\tau$. For the viscoelastic modes, the stress contribution $\varvec{\tau }_{\mathrm{ve},i}$ is obtained using the kinematics as previously described (van Dam et al. 2008). In short, for a new time-step, the deformation gradient tensor $\varvec{F}$ and Eq. (9) are used to update the right Cauchy-Green tensor $\varvec{C}_{\mathrm{p},i}$, which then gives a new elastic deformation tensor $\varvec{B}_\mathrm{e}$ using Eq. (8). The viscoelastic stress $\varvec{\tau }_{\mathrm{ve},i}$ is then obtained using constitutive Eq. (11) and is used to update the inelastic rate of deformation tensor $\varvec{D}_{\mathrm{p},i}$ [Eq. (12)] that is used in the next step to update $\varvec{C}_{\mathrm{p},i}$.

Parameter values are found using a stepwise fitting procedure. A flowchart of this procedure is provided as supplementary material, Figure S1.

The first step of the procedure is to determine the linear viscoelastic parameters $G_{i0}$ and $\eta _{i0}$ using the results of the frequency sweep and Eqs. (15) and (16). These quantities are then fixed and used for the description of the time-dependent behavior during the formation of the clots and to determine the corresponding values of the parameters $t_\mathrm{c}$ and $t_0$. The linear viscoelastic parameters also serve as input for the fitting procedure of the nonlinear viscoelastic part of the model.

First, for every cycle of the LAOS measurement, the maximum strain $\gamma _0$, corresponding stress $\tau _0$, the minimal strain modulus $G_m$ and the stress at a strain of $\frac{1}{2}\, \gamma _0$ and $\frac{\sqrt{2}}{2}\, \gamma _0$ are determined. The next step is to determine the parameters that describe the softening and the strain-stiffening effects, *a*, *c* and $k_1, n_1$, respectively. A complication is that both the softening and the stiffening behaviors are influenced by both parameter sets. This can be seen by the stress at maximum strain $\tau _0$, that is a function of the softening parameter $x_i$ and the strain-stiffening function $f_\mathrm{ss}$ [see Eq. (26)]. Therefore, the parameters that describe the softening, *a* and *c*, influence the values of the stiffening parameters $k_1$ and $n_1$, and vice versa, and an iterative procedure is needed to find parameter values. Given values for *a* and *c*, the strain-stiffening parameters $k_1$ and $n_1$ are determined using Eq. (27). These values are then used to determine the stress at maximal strain, $\tau _0$ and to find parameter values for the softening parameters *a* and *c*, which can then be used in the next iteration step. This iteration cycle is continued until the relative change in parameter values is $<$0.01. The iterative procedure is initiated using an initial guess for *a* and *c*, obtained by fitting the behavior of the low strain modulus $G_m$/$G_m\left( 0\right)$ using the evolution equation for $x_i$ (see Fig. 6a).

The last step of the fitting procedure is to determine the parameter $k_2$ that describes the nonlinear viscous dissipation. The value of this parameter is found using the stress at $\frac{1}{2}\, \gamma _0$ and $\frac{\sqrt{2}}{2}\, \gamma _0$, because at these moments, the viscous contribution to the stress and the stress itself are relatively large (van Kempen et al. 2015).

An overview of the entire fitting procedure is provided as supplementary material, Figure S1. For every step, an objective function is minimized that represents the difference between experimentally determined or estimated values, denoted with a subscript e, and values given by the model. All minimizations are performed using the nonlinear least squares solver *lsqnonlin* with the trust-region-reflective algorithm as implemented in the Global Optimization Toolbox of MATLAB (The MathWorks, Natick, MA, USA). All minimizations are performed multiple times with different initial values to avoid that a local minimum is found.

To assess the quality of the fitting procedure, a mean relative error is defined that quantifies the difference between the experimentally and numerically determined stress,

43 $$\begin{aligned} \zeta = \frac{1}{N} \sum _{n=1}^N \left| \frac{ \tau _n^e - \tau _n^m}{\tau ^m_n}\right| , \end{aligned}$$

with *N* being the number of time steps and $\tau _n^e$ and $\tau _n^m$ the stress at point *n* as determined in the experiment and from the model, respectively.

### Sensitivity analysis

To study the influence of a variation in parameter values to the output of the model, a sensitivity analysis is performed. This analysis is focused on the part of the model that describes the nonlinear viscoelastic behavior and its five parameters. The analysis applied here is based on a global variance-based method (Sobol 2001; Saltelli 2002; Huberts et al. 2014) and is an extension of a previously applied analysis (van Kempen et al. 2015). The method estimates the contribution of a parameter due to its uncertainty to the variance of a defined output of the model. These contributions to the output variance are given in terms of two sensitivity indices for every parameter. The main sensitivity index $S_i$ is the relative contribution of parameter *i* to the total variance of an output. This index quantifies the direct influence of a variation in the parameter on an output. A second index, the total sensitivity index, $S_i^\mathrm{T}$ quantifies this main effect plus indirect contributions of parameter *i* to the total variance of an output by interactions with all other parameters. A small $S_i^\mathrm{T}$ indicates that the parameter has a small contribution to the variance of an output and might be fixed within its uncertainty domain (Huberts et al. 2014).

The sensitivity analysis estimates the contribution of a parameter to a certain output of the model. These outputs have to be defined and are chosen the same as used previously for a model for the nonlinear viscoelastic properties of the fibrin network (van Kempen et al. 2015). The three outputs chosen describe the three nonlinear features of the model. The first output, $O_\mathrm{so}$, is related to the softening effect and is defined as the relative decrease in $\tau _0$ during the strain interval with amplitude $\gamma _0 = 1$. The second output, $O_\mathrm{ss}$, is related to strain stiffening and is defined as the maximum stress during the aforementioned strain interval, normalized with the linear equilibrium modulus $G_{00}$. A third output is related to the nonlinear viscous dissipation and is defined as the width of the first Lissajous–Bowditch cycle of the same strain interval, at a strain of $\gamma = \frac{\sqrt{2}}{2}\gamma _0$.

The sensitivity indices can be determined using a Monte Carlo method (Saltelli 2002), but this requires at least $10^3$ model runs per parameter. A more efficient method has been proposed recently (Crestaux et al. 2009; Huberts et al. 2014) and is used here. In short, a relatively small number of model runs are used to sample the output space. This space is subsequently described using a metamodel that consists of polynomials that are a function of the input parameters. This so-called generalized polynomial chaos expansion is then used to efficiently determine the sensitivity indices. More details about this method can be found elsewhere (Huberts et al. 2014).

For every output, the main and total sensitivity indices are determined for every parameter. The analysis is based on 280 model runs, which is five times the required minimum for a model with five parameters (Huberts et al. 2014). The input parameters are drawn from a specified range using quasi-random Sobol sequence (Sobol 2001). The parameter range is the mean $\pm$ standard deviation of the values obtained by fitting the model to four data sets, as shown in Sect. 3.

## Results

In this section, the results generated using the model are presented. The results for clots formed from blood from one pig are presented in detail, while the parameter values of the model are shown as means with standard deviations as obtained from the blood of four pigs.

### Frequency response

**Fig. 8:**
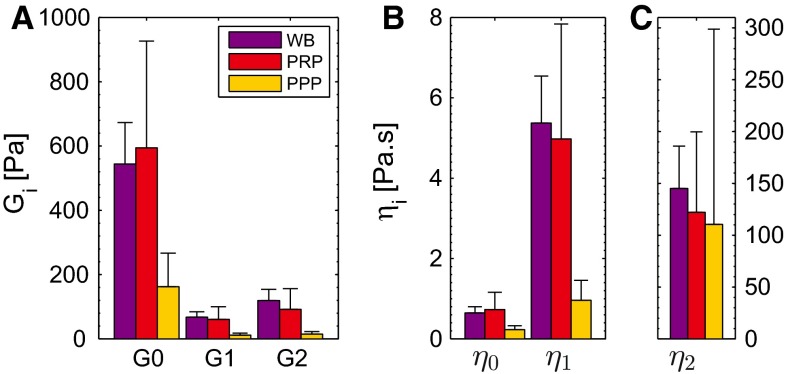
The moduli $G_i$ (**a**) and viscosities $\eta _i$ (**b**, **c**), for clots with different compositions. Values are shown as mean + standard deviation of four data sets

As already shown in Fig. 3a, the viscoelastic model describes the frequency response of the different clots well, using a total of six linear viscoelastic parameters. The values of the elastic modulus $G^{\prime }$ are at least an order of magnitude larger than of the viscous modulus $G^{\prime \prime }$, which shows that the clots behave as viscoelastic solids. This is supported by the minor frequency dependency of the moduli, i.e., the absence of a terminal zone. In agreement with this are the high values of the modulus of the equilibrium mode, as seen in Fig. 8a. For clots formed from PRP, the mean values of the moduli are slightly higher than for those formed from WB, while the values for clots formed from PPP are much lower than for those of WB and PRP. Due to the lower moduli, the viscoelastic modes have a relatively small, but significant contribution in comparison with the equilibrium mode with modulus $G_0$. The viscosities of the Maxwell modes are much higher than $\eta _0$ and are lowest for clots formed from PPP (Fig. 8b, c).

### Blood clot formation

**Fig. 9:**
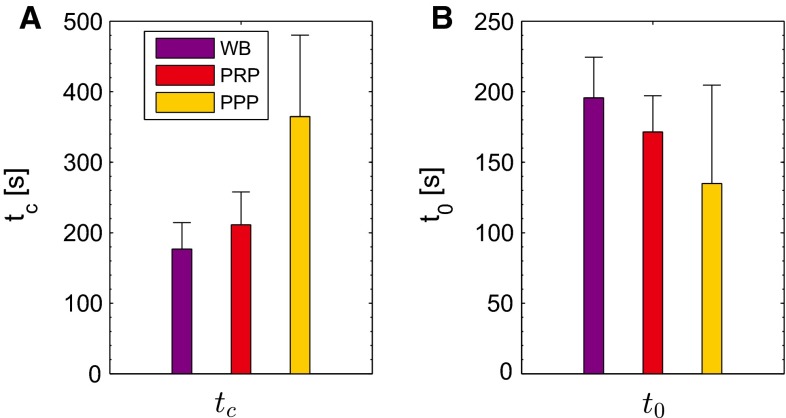
The parameters that describe the time dependency of the mechanical properties of the developing clots. The time constant $t_\mathrm{c}$ (**a**) and delay time $t_0$ (**b**) are shown as mean + standard deviation of four data sets

The moduli and viscosities, obtained from the frequency response, are made time dependent to describe the mechanical properties of the clots during their formation. The results for a clot formed from WB are shown in Fig. 3b–d. The viscoelastic moduli of the thrombi increase to a steady value within 30 min. The model describes the increase in the elastic modulus $G^{\prime }$ (b) and viscous modulus $G^{\prime \prime }$ (c) well, and also their ratio as shown by the decreasing phase angle $\delta$ (d). The model accurately describes the transition from fluid-like to solid-like behavior. The corresponding parameter values are shown in Fig.  9. The time constant $t_\mathrm{c}$ increases, and the delay time $t_0$ decreases for clots formed from WB, PRP and PPP. This indicates that the transition from fluid to solid starts earlier for the clots formed from PPP and propagates at a slower rate.

**Fig. 10:**
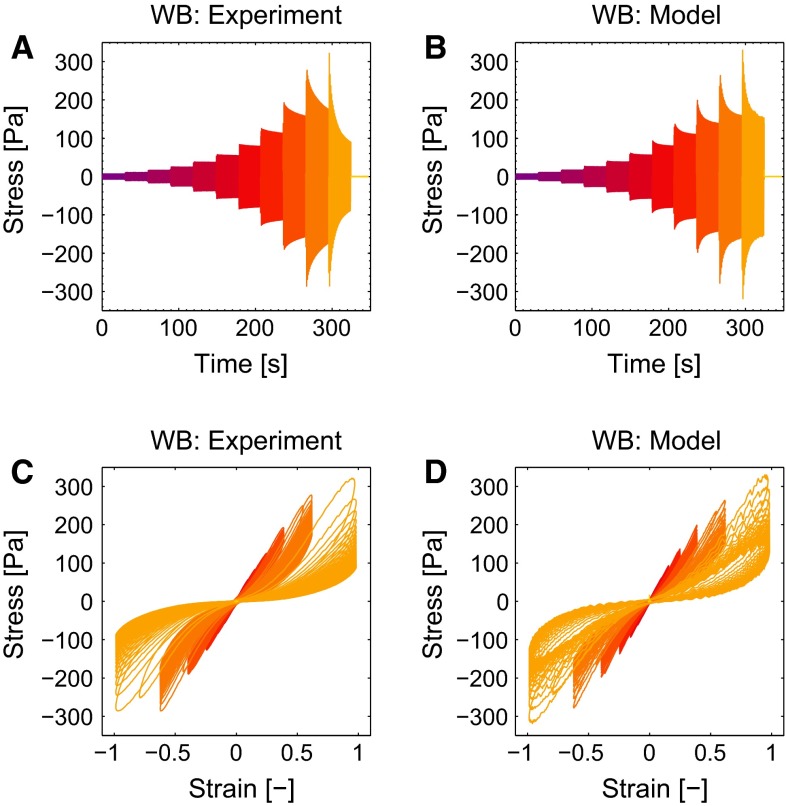
The stress in time as experimentally measured (**a**) and predicted by the model (**b**) for a blood clot formed from whole blood agrees well. The Lissajous–Bowditch plots show that the model (**d**) describes the experimentally observed nonlinear behavior (**c**)

### Nonlinear viscoelastic behavior

**Fig. 11:**
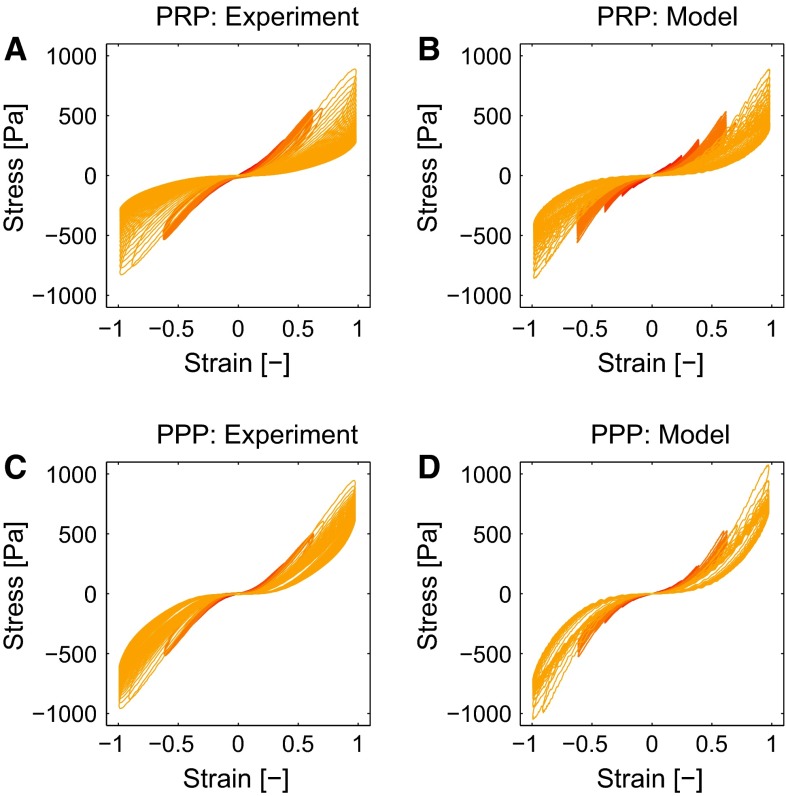
The Lissajous–Bowditch plots for clots formed from PRP (**a**, **b**) and PPP (**c**, **d**) as measured experimentally (**a**, **c**) and described by the model (**b**, **d**)

**Fig. 12:**
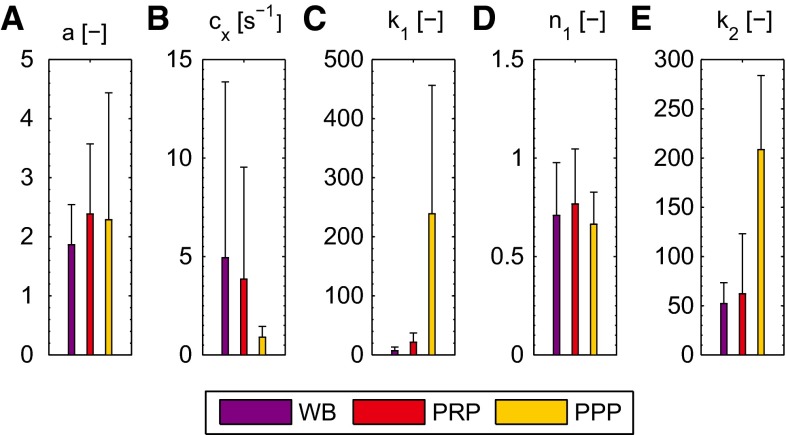
Values for the softening parameters *a* (**a**) and $c_x$ (**b**), the strain-stiffening parameters, $k_1$ (**c**) and $n_1$ (**d**) and the viscous dissipation parameter $k_2$ (**e**) for clots with different compositions. Values are shown as mean + standard deviation for four data sets

The results of the LAOS experiments that probe the nonlinear viscoelastic behavior are shown for a clot formed from WB in Fig. 10. When observing the stress in time (a and b), it can be seen that the maximal stresses that occur during the cycles are well captured by the model. The three nonlinear features, softening, strain stiffening and nonlinear viscous dissipation, are clearly visible in the Lissajous–Bowditch plots of the experiment (c), as well as in the model (d). For the largest strain amplitude of $\gamma _0 = 1$, the model overestimates the viscous dissipation, which is most likely caused by the quickly decreasing stress during this interval due to structural damage of the clot. For the lower strains, the description is better. Overall, the stress response is described by the model with a mean relative error, $\zeta$, of 0.10. The Lissajous–Bowditch plots for clots formed from PRP and PPP are shown in Fig. 11. Qualitatively the same behavior is visible as seen for clots formed from WB, but the maximal stresses are about three times as high. The softening behavior is less pronounced for the clots formed from PRP and PPP. The model describes this behavior well, as shown by a mean relative error $\zeta$ of 0.15 and 0.16 for the thrombi formed from PRP and PPP, respectively. The values of the parameters used to describe the nonlinear viscoelastic behavior are shown in Fig. 12. From the two parameters related to the softening, *a* and $c_x, a$ does not differ considerably for the different clots, while the value for $c_x$ is lower for clots formed from PRP and PPP albeit with large standard deviations. This indicates that the stiffness of those clots decreases faster, but that the value to which it decreases is the same for the different clots.

The clots formed from PPP show the largest amount of strain stiffening, as indicated by the high value of the parameter $k_1$. Clots formed from PRP show less strain stiffening. They reach the same maximal stress during the LAOS deformation as the clots formed from PPP, but their linear viscoelastic stiffness is higher, which leads to a lower value for $k_1$. The values of the other parameter related to strain stiffening, $n_1$, do not differ remarkably with clot composition.

The single parameter related to the nonlinear viscous dissipation is $k_2$. It has a higher value for clots formed from PPP than for those formed from PRP and WB, i.e., the nonlinear viscous dissipation is more pronounced.

**Fig. 13:**
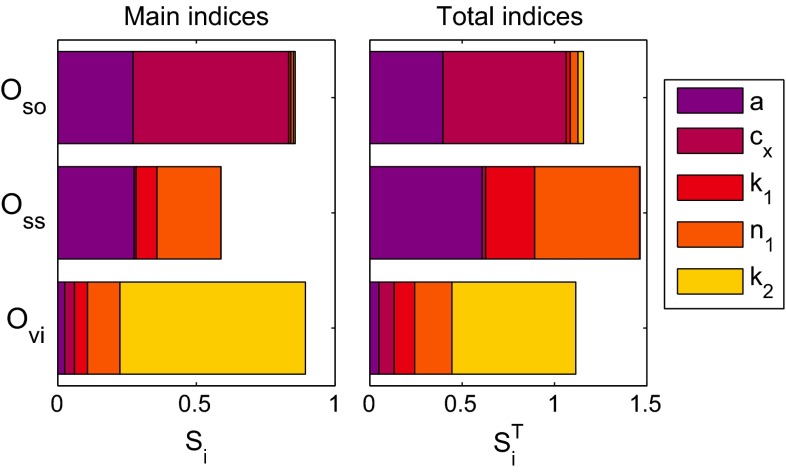
The main (*left*) and total (*right*) indices obtained from a sensitivity analysis for the outputs describing softening $(O_\mathrm{so})$, strain stiffening $(O_\mathrm{ss})$ and nonlinear viscous dissipation $O_{\mathrm{v}i}$

### Sensitivity analysis

The results of the sensitivity analysis are shown in Fig. 13. The variance of the output that describes the softening behavior $O_\mathrm{so}$ is, as expected, almost completely determined by the parameters *a* and $c_x$. The output related to strain stiffening, $O_\mathrm{ss}$, is besides the parameters $k_1$ and $n_1$ also influenced by the parameter *a* that relates to the softening behavior. For this output, the sum of the total indices is larger than one, which is an indication that interactions between parameters contribute to the total variance (Huberts et al. 2014).

The variance of the output related to the viscous dissipation, $O_{\mathrm{v}i}$, is dominated by the parameter $k_2$, which is expected. However, also the parameters related to strain stiffening, $k_1$ and $n_1$, have important contributions.

## Discussion

The constitutive model developed consists of different parts that describe the viscoelastic properties of the clots during their formation, during a frequency sweep and during LAOS. The model correctly captures these mechanical properties during the various deformations for clots formed from WB, PRP and PPP using specific parameter values.

The rheological results show that the stiffness of clots formed from WB is slightly lower than of those formed from PRP, while those formed from PPP have a much lower stiffness (Fig. 3). These findings are in agreement with previously reported results (Kaibara and Fukada 1970; Glover et al. 1975; Kirkpatrick et al. 1979; Jen and McIntire 1982; Shah and Janmey 1997; Riha et al. 1999; Tynngård et al. 2006). The explanation for this behavior is that the contracting platelets increase the stiffness of the fibrin network by pulling on the fibers (Jen and McIntire 1982; Lam et al. 2011). The high volume of red blood cells in WB prevents this by distorting the clot formation and platelet contraction (Riha et al. 1999; Tynngård et al. 2006; Gersh et al. 2009). This also explains why the difference between clots formed from PRP and WB is larger during the LAOS experiments (Figs. 10, 11); the fibrin network is stiffer when there are no red blood cells present that prevent a regular network formation and platelet binding. This also shows that the analysis of the nonlinear viscoelasticity in terms of Lissajous–Bowditch provides more insights than the traditionally used linear viscoelastic moduli (Shah and Janmey 1997; Riha et al. 1999).

The influence of clot composition is incorporated in the model by adjusting the parameter values. This rather simple phenomenological description gives satisfying results, but could be extended by relating the parameters to the concentration of the individual components. This would be especially useful if the model is going to be applied to simulations of blood clot formation, in which the concentration of the components are known (Bodnár and Sequeira 2008; Storti et al. 2014). However, an advantage of the current description is that different clot compositions can be studied by simply changing parameter values. This is useful for numerical simulations that often describe parts of the clotting system (Anand et al. 2003; Bodnár and Sequeira 2008; Xu et al. 2011; Storti et al. 2014). Furthermore, the model is relatively simple and can easily be extended to describe the mechanical behavior of materials showing similar behavior such as collagen (Münster et al. 2013), keratin filaments (Ma et al. 1999), gluten gel (Ng et al. 2011) and skin (Lamers et al. 2013). A possible extension would be to explicitly incorporate structural clues that underlie the nonlinear behavior, such as the behavior of the fibrin fibers during strain stiffening (Münster et al. 2013).

Values of the parameters are found using a stepwise fitting procedure. This procedure does not ensure that the best parameter set is obtained, because it omits possible interactions between parameters. The applied sensitivity analysis provides useful insights into this. The analysis shows that the softening behavior is influenced almost completely by the parameters related to this phenomenon, *a* and $c_x$. The strain-stiffening behavior is influenced not only by its related parameters $k_1$ and $n_1$, but also by the softening parameter *a*. Lastly, the nonlinear viscous dissipation is influenced by all parameters. This shows that it makes sense to start the iterative procedure between the softening and stiffening effects with the former and also to fit the viscous parameter $k_2$ as the last step. As all parameters contribute to the total indices of one ore more outputs, the model cannot be simplified by fixing one of them within its uncertainty interval.

The parameter values found in this study are based on clots formed from four pigs. Although the qualitative behavior of the clots is the same, large variations in parameter values are present. This shows that it is difficult to make quantitative predictions about the mechanical properties of an individual. Despite these variations, the model is able to describe the different clots.

Although the model is suitable to describe any arbitrary deformation, it is only tested against rheological experiments in which a simple shear deformation is applied. The LAOS deformation is expected to mimic the situation in a pulsatile blood flow, but nevertheless other deformations such as compression (Kim et al. 2014) and extension (Brown et al. 2009) will be useful to further test the model.

## Conclusion

This study presents a constitutive model for blood clots with different compositions. The model describes the mechanical properties during the formation of the clot, its frequency response and its time-dependent, nonlinear viscoelastic properties. Clots with various compositions, i.e., fibrin, platelets and red blood cells, are formed and described by the model using different parameter values. Despite its relative simplicity, the model is able to describe the complex behavior of the blood clots.

## Electronic supplementary material

Supplementary material 1 (docx 178 KB)
