# Supplementary material for: A constitutive model for developing blood clots with various compositions and their nonlinear viscoelastic behavior
Source: Biomech Model Mechanobiol. 2015 Jun 5;15:279–91. doi: 10.1007/s10237-015-0686-9 (PMC4792371; doi:10.1007/s10237-015-0686-9)
Supplement: Supplementary file 1 — Supplementary material 1 (docx 178 KB) [file 10237_2015_686_MOESM1_ESM.docx]

**Supplementary material
A constitutive model for developing blood clots with various compositions and their nonlinear viscoelastic behavior**Thomas H.S. van Kempen*, Wouter P. Donders , Frans N. van de Vosse, Gerrit W.M. Peters

*Corresponding author: Department of Biomedical Engineering, Eindhoven University of Technology, [t.h.s.v.kempen@tue.nl](mailto:t.h.s.v.kempen@tue.nl)

**S1. Flowchart of the parameter optimization process**
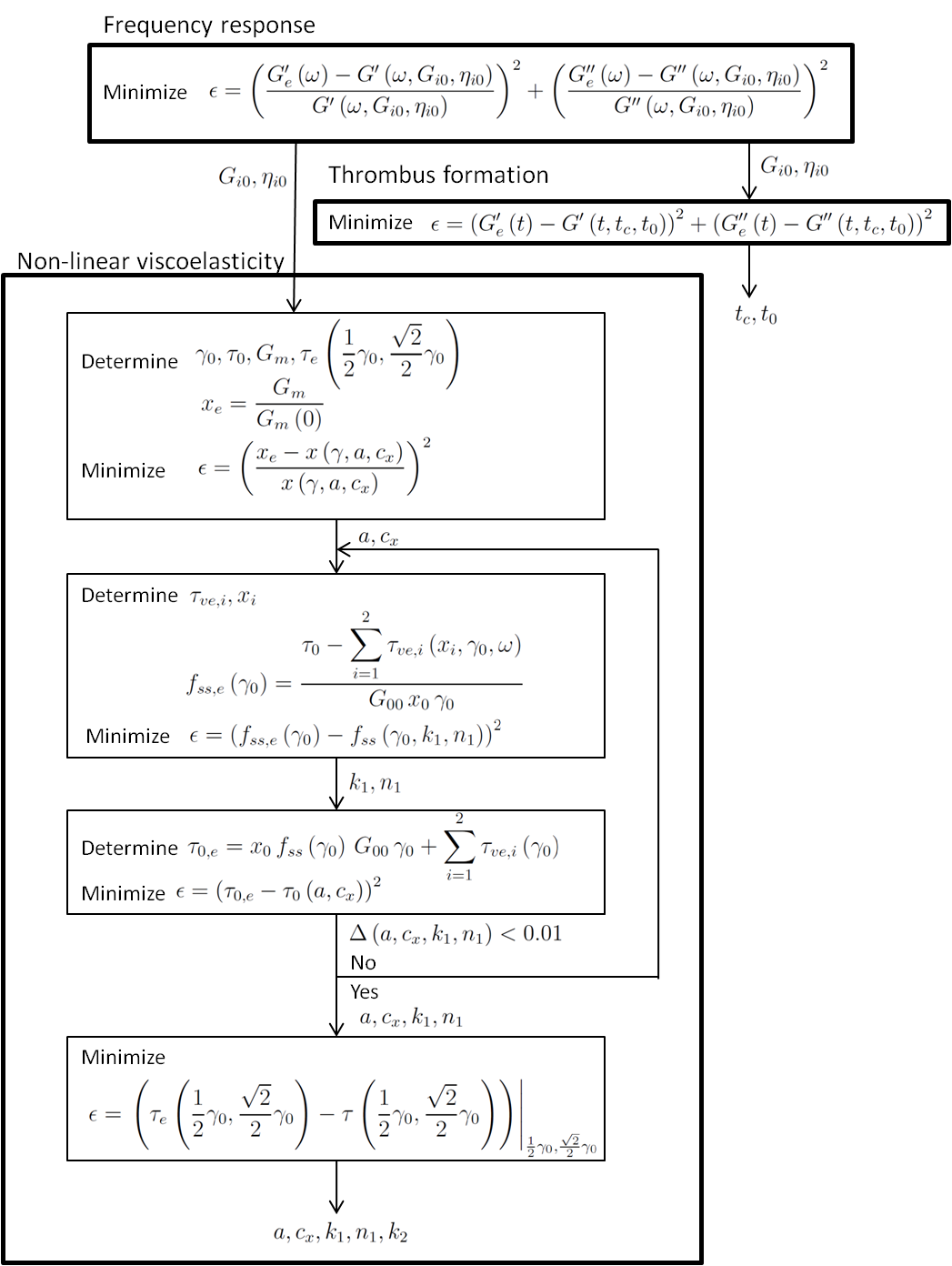


Figure S1 Flowchart of the procedure to obtain model parameters.
